# Differential lipids in euthyroid pregnant women with positive TPOAb and its correlation with clinical parameters

**DOI:** 10.3389/fendo.2025.1433534

**Published:** 2025-03-27

**Authors:** Xinxin Chen, Yuxin Qin, Qingyao Wang, Ying Wu, Huanhuan Zang, Xiangguo Cong, Qiong Shen, Lei Chen

**Affiliations:** Department of Endocrinology, The Affiliated Suzhou Hospital of Nanjing Medical University, Suzhou, China

**Keywords:** thyroid peroxidase antibody, pregnancy, lipids, metabolism, thyroid function

## Abstract

**Introduction:**

Pregnant women with subclinical hypothyroidism or clinical hypothyroidism often exhibit lipid metabolism disorders and are correlated with adverse pregnant outcomes. It was suggested that isolated positive thyroid peroxidase antibody (TPOAb) served as a risk factor for adverse outcomes. However, little was known about the lipid metabolism profile in pregnant women with isolated positive TPOAb. The purpose of this prospective observational study was to investigate the expression of lipid profiles among euthyroid pregnant women with positive TPOAb during there early pregnancy and to analyze their correlation with thyroid function.

**Methods:**

Non-targeted liquid chromatography-mass spectrometry (LC-MS) technology was used to perform lipidomics analysis on serum samples collected during early pregnancy from pregnant women who with isolated positive TPOAb and those in the healthy control group. Partial least squares-discriminant analysis (PLS-DA), KEGG (Kyoto Encyclopedia of Genes and Genomes) pathway enrichment analysis, and correlation analysis were conducted to explore differential lipid molecules and their associations with clinical parameters.

**Results:**

A total of 90 pregnant women in the first trimester were enrolled in the analysis: 46 were TPOAb-positive euthyroid pregnant women, and 44 were healthy pregnant women. A total of 1238 lipid molecules were identified, and 202 differential lipid molecules were screened between the two groups. KEGG pathway enrichment analysis revealed that the differentially expressed lipids participate in several pathways. Correlation analysis showed LPC(20:4), LPC(18:0), LPC(22:4), LPC(22:5), LPC(18:1), PC(20:1/20:4) were both positively correlated with TPOAb titers and sCD40L. LPC(20:0) was positively correlated with the level of remnant cholesterol (RC) and PC(20:1/20:4) was negatively correlated with RC.

**Discussion:**

The lipid profile of isolated TPOAb-positive euthyroid pregnant women was significantly different from that of healthy pregnant women and involved in several pathways. The pathophysiological role of altered lipid molecules should be further investigated since they might be potential biomarkers for adverse pregnancy outcome in pregnant women with isolated positive TPOAb.

## 1 Introduction

Autoimmune thyroid disease is the most common endocrine disorder affecting about 8-14% women of child-bearing age (1). During pregnancy, the presence of circulating TPOAb/TgAb autoantibodies or insufficient thyroid hormone (such as clinical or subclinical hypothyroidism) has the potential to disrupt the equilibrium of thyroid hormones which can adversely influence fetal development (2, 3).

Isolated TPOAb positivity, defined as the detection of TPOAb and thyroid hormones within the reference range during pregnancy, was found in nearly 4% to 15% of pregnant women (4). Numerous studies have shown that isolated TPOAb contributes to the development of adverse pregnancy and neonatal outcomes, such as recurrent spontaneous abortion, gestational diabetes mellitus, premature delivery, and fetal growth restriction (5, 6). However, the underlying mechanism and potential risk factors remained unclear. In our previous study, isolated TPOAb-positive pregnant women were shown to have a greater risk of gestational diabetes mellitus than TPOAb-negative women, as indicated by increased triglyceride (TG) and soluble CD40 ligand (sCD40L) levels in the serum (7, 8). In addition, the specific treatment goal or intervention for these special phenotype women to reduce the risk of adverse pregnant outcome remained ambiguous since the thyroid parameters is normal (9, 10).

Thyroid hormones have significant effects on various aspects of lipid metabolism, including enhancing the utilization of lipid substrates, mobilizing TG stored in adipose tissue, and augmenting the activities of hepatic lipase (HL) and cholesterol transfer protein (CETP). It is well established that dyslipidemia is a common feature of thyroid dysfunction, and hypothyroidism often manifested with increased levels of low-density lipoprotein cholesterol (LDL-c), TG, and total cholesterol (TC) (11, 12). Disorders of maternal lipid metabolism have been shown to be associated with an increased risk of multiple adverse pregnancy outcomes including gestational diabetes mellitus, gestational hypertension, preeclampsia, and preterm birth (13). Thus, lipid molecules might act as a bridge between thyroiditis and adverse outcome during pregnancy.

By using liquid chromatography-mass spectrometry (LC-MS), lipidomic study represents an emerging discipline that hold great potential in revealing the association between lipid biology and disease. Shao et al. reported that pregnant women with subclinical hypothyroidism (SCH) and clinical hypothyroidism (CH) had significantly greater plasma glycerophospholipid levels than that in healthy individuals, while there were no significantly changed metabolites between the CH group and the SCH group (14). Some metabolites exhibited a similar pattern in Hashimoto thyroiditis (HT) patients with hypothyroidism compared to healthy controls, suggesting that thyroid hormone levels are not the only influencing factor for changes in serum metabolites and that thyroid autoimmunity may also be involved (15, 16). And more importantly, changes of thyroid hormones might not be able to reflect the metabolite changes accurately. In non-pregnant women, epidemiological studies have revealed the emerging role of lipid disorders and the development of hypothyroidism (17, 18). It was speculated that TPOAb-positive pregnant women may exhibit distinct lipid metabolic abnormalities and lipidemia may not necessarily be a consequence of thyroid dysfunction but also that thyroid dysfunction may arise from lipid metabolism disorders.

However, few studies concentrated on lipid metabolism in isolated TPOAb-positive euthyroid pregnant women. It remained unknown that whether isolated TPOAb-positive euthyroid pregnant women accompanied with dysregulated lipid profile. This study aimed to provide a comprehensive evaluation and comparison of the metabolic changes in isolated TPOAb-positive pregnant women with normal thyroid function using LC-MS.

## 2 Participants and methods

### 2.1 Participants

The study was designed as a prospective observational study. Pregnant women who underwent routine prenatal examination in Suzhou Hospital Affiliated to Nanjing Medical University from March 2020 to July 2021 were enrolled as participants in this observational study. A total of 46 TPOAb-positive euthyroid pregnant women and 44 TPOAb-negative euthyroid healthy controls, who met the specified criteria, were included in the study.

Inclusion criteria were as follows: (1) Thyroid function levels during pregnancy were within the normal range, specifically thyroid stimulating hormones (TSH) levels of 0.35~4.94 μIU/ml and free thyroxine (FT4) levels of 9.01~19.04 pmol/L. Positive TPOAb status was defined as having TPOAb levels ≥ 12 IU/ml. (2) All participants were in their first trimester of pregnancy.

Exclusion criteria: (1) Age >35 years. (2) Missing data/records. (3) Previous history of thyroid-related disease. (4) Taking drugs that affect thyroid function or undergoing immuno-suppressive therapy. (5) Personal history of obesity, metabolic syndrome or gestational diabetes. (6) Personal history of chronic systemic disease. (7) Personal history of hereditary diseases. (8) Recent infectious disease. (9) To eliminate potential effect of subtle thyroid dysfunction due to positive TPOAb, TSH>2.5 µIU/ml in isolated TPOAb positivity pregnant women were also excluded (9).

Inform consents were obtained from participants prior to their enrollment in the study. The study was approved by the Ethics Committee of the affiliated Suzhou Hospital of Nanjing Medical University (approval number: K-2020-063-K01).

### 2.2 Data collection and laboratory measurements

The demographic data of all participants were recorded in the first trimester, including age, gestational weeks, gravida, parity, and pre-pregnancy body mass index (BMI). All participants underwent routine obstetric examinations at Suzhou Hospital Affiliated to Nanjing Medical University. Clinical data were collected, including triglycerides (TG), total cholesterol (TC), high-density lipoprotein cholesterol (HDL-c), low-density lipoprotein cholesterol (LDL-c), glycated hemoglobin A1c (HbA1c), ferritin levels and uric acid (UA). Fasting remnant cholesterol (RC) was calculated as TC − (HDL-c + LDL-c) (mmol/L).

Automated chemiluminescent immunoassays (Architect i2000SR instrument from Abbott Laboratories, Chicago, IL) were utilized to measure serum levels of TSH, FT4, and TPOAb. Concentrations of serum soluble CD40 ligand (sCD40L) were measured using ELISA kits (Xvguang Kexing Antibody Biotechnology Co., Ltd). Both intra-assay and inter-assay coefficient of variation for sCD40L were maintained at less than 5.00% to ensure accuracy and reliability. To minimize assay variability, all samples were tested in the same batch. Following data collection and laboratory measurements, we analyzed differential metabolites using supervised statistical methods and pathway enrichment analysis.

### 2.3 Major instruments and reagents

Instruments used in this study included an ultrahigh-performance liquid chromatograph (UPLC) (Vanquish Horizone, Thermo Fisher Scientific, USA), a high-resolution mass spectrometer (Q Exactive HF-X quadrupole-Orbitrap, Thermo Fisher Scientific, USA), a chromatographic column: Accucore C8 (2.6μm, 2.1×100 mm, Thermo Fisher Scientific, USA), a low-temperature ultracentrifuge (Centrifuge 5430R, Eppendorf), a water purifier (Milli-Q Integral, Millipore Corporation, USA), and a refrigerated vacuum concentrator (LNG-T88, HuaMei instrument, China). Reagents used in this study included MTBE (methy tert-butyl ether, Adamas-beta company, China), LC-MS-grade methanol (Thermo Fisher Scientific, USA), LC-MS-grade acetonitrile (Thermo Fisher Scientific, USA), LC-MS-grade isopropanol (Thermo Fisher Scientific, USA), LC-MS-grade ammonium formate (Thermo Fisher Scientific, USA), LC-MS-grade formic acid (Thermo Fisher Scientific, USA) and water purified by a water purifier.

### 2.4 Specimen collection and UHPLC-MS analysis

Both groups were in their first trimester. To reduce the possible effects of hormone fluctuations and changes of dietary habits during pregnancy and to extend the observational time, we collected the samples during the first admission of these pregnant women when they firstly registered for prenatal care. All participants fasted for 8-12 hours before intravenous blood collection. Serum sample was stored in -80°C refrigerator before centralized measurement. Lipid molecules were extracted from the specimen and a mixed quality control (QC) sample was prepared.

LC-MS/MS analysis of samples was performed using a Thermo UHPLC-Q Exactive HF-X Vanquish Horizon system with an Accucore C8 column at Majorbio Bio-Pharm Technology Co. Ltd. Samples were maintained at 4°C throughout the analysis to ensure stability. The mass spectrometric data was gathered using a Thermo UHPLC-Q-Exactive HF-X Benchtop Orbitrap Mass Spectrometer, featuring a heated-electrospray ionization (HESI) source operating in positive and negative ion mode. Data acquisition was performed in Data-Dependent Acquisition (DDA) mode. After UPLC-MS/MS analysis, raw data were imported into the LipidSearch (Thermo, CA) for peak detection, alignment, and identification. Lipid molecules observed in 80% of the samples were retained in the final analysis, and variables with relative standard deviation (RSD) > 30% of QC samples were removed. Later, R package (Version 4.3.1) is used for differential metabolites analysis.

### 2.5 Differential metabolites analysis

All statistical analyses were performed by SPSS 25.0, and the results were considered statistically significant with values of *P* < 0.05. In the clinical data of the two groups, the normal distributions were expressed by mean ± standard deviation, and the non-normal distributions were described with 95% confidence intervals. Non-parametric tests were used to analyze the differences between groups.

A combination of the Variable importance in the projection (VIP) obtained by the supervised partial least squares-discriminant analysis (PLS-DA) and the p-value of student’s t-test was used to screen differential metabolites between groups. The metabolites with VIP > 1, *P* < 0.05 were significantly different metabolites.

Differential metabolites between the two groups were summarized and mapped into their biochemical pathways through metabolic enrichment and pathway analysis based on database search (Kyoto Encyclopedia of Genes and Genomes (KEGG), http://www.genome.jp/kegg/). R package of ggsankey (https://github.com/davidsjoberg/ggsankey) was exploited to identify statistically significantly enriched pathway. R package of ggplot2 (https://ggplot2.tidyverse.org) was used to visualize analysis of significance correlations between lipids molecules and clinical parameters using Spearman’s correlation analysis. Correlation coefficients (*r*) and *P* value were calculated. *P* < 0.05 was considered statistically significant.

## 3 Results

### 3.1 General characteristics of the study population

This observational study enrolled 46 euthyroid pregnant women with isolated TPOAb positivity and 44 healthy pregnant women as controls during the first trimester of pregnancy. There were no significant differences in age, pre-pregnancy BMI, gravity, parity, TSH, FT4, ferritin, HbA1c or TG between the 2 groups. However, the serum LDL-c and TC concentration was significantly lower while RC was significantly greater in the group of isolated TPOAb-positive euthyroid pregnant women (*P* < 0.05) (showed in **Table 1**).

**Table 1:** The comparison of baseline and laboratory parameters of participants between TPOAb negativity and TPOAb positivity in early pregnancy.

Variables	TPOAb negativity(n=44)	TPOAb positivity(n=46)	*P* value
Age	29.49 ± 3.80	30.15 ± 3.71	0.386
Pre-pregnancy BMI	22.33 ± 2.91	22.17 ± 2.78	0.786
Gravida	2 (1-2)	2 (1-3)	0.490
Parity	0 (0-1)	0 (0-1)	0.417
TSH (µIU/ml)	1.25 ± 0.89	1.37 ± 0.62	0.062
FT4 (pmol/L)	13.43 ± 2.21	12.91 ± 1.32	0.217
Ferritin (ng/ml)	72.76 ± 46.42	64.13 ± 38.36	0.407
HbA1c (%)	5.24 ± 0.23	5.22 ± 0.24	0.991
UA (µmol/L)	228.50 ± 57.13	223.41 ± 46.43	0.293
TG (mmol/L)	1.49 ± 0.57	1.28 ± 0.56	0.481
**TC (mmol/L)**	**4.77 ± 0.90**	**4.55 ± 0.58**	**0.004**
HDL-c (mmol/L)	1.78 ± 0.35	1.77 ± 0.32	0.317
**LDL-c (mmol/L)**	**2.58 ± 0.77**	**2.26 ± 0.55**	**0.019**
**RC(mmol/L)**	**0.41 ± 0.24**	**0.50 ± 0.15**	**0.002**

*P* < 0.05 is statistically significant. Bold values in the table indicate statistically significant differences between groups (*P* < 0.05).

BMI, body mass index; TPOAb, thyroid peroxidative antibodies; TSH, thyroid stimulating hormones; FT4, free thyroxine; HbA1c, glycosylated hemoglobin A1c; UA, uric acid; TG, triglyceride; TC, total cholesterol; LDL-c, low density lipoprotein cholesterol; HDL-c, high density lipoprotein cholesterol; RC, remnant cholesterol, calculated with the formula.

### 3.2 Differential lipid analysis between groups

**Figure 1:**
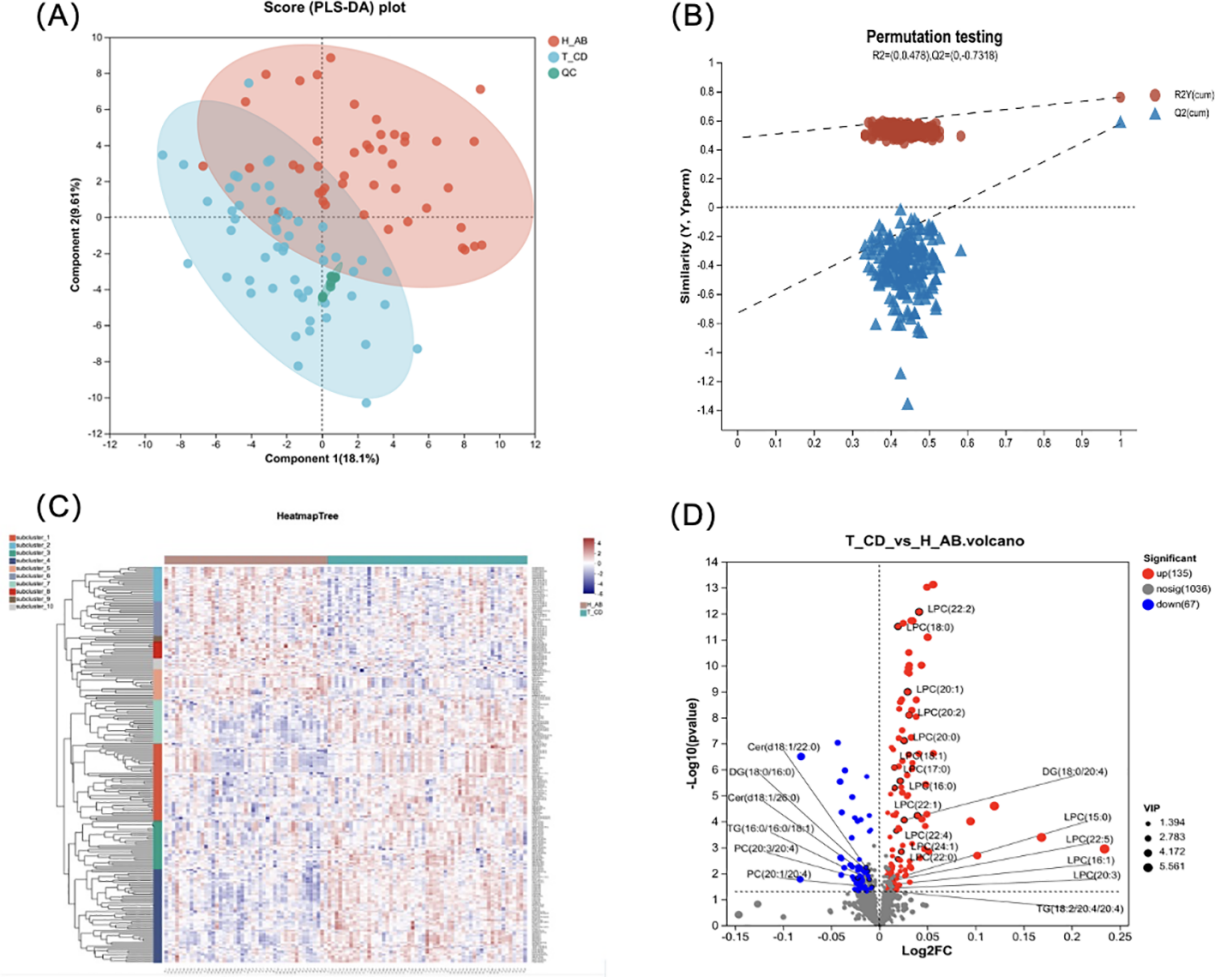
Difference analysis for the isolated positive TPOAb women and healthy control during pregnancy. **(A)**The PLS-DA model score plot showed a significant difference between 2 groups. (2 Principal components, component 1 score: 18.1%, component 2 score: 9.61%); **(B)** Model verification map of PLS-DA analysis: R2 = (0.0, 0.478), Q2 = (0.0, -0.7318). **(C)** The cluster analysis diagram shows the aggregation trend in lipid profile between the isolated positive TPOAb group and control groups. The different shades of color indicate the intensities, of which the blue indicates low-intensity and red high-intensity). **(D)** Volcano plot shows the number of dysregulated lipids in the isolated positive TPOAb group compared to the control group. 25 lipid molecules were marked in the plot.

PLS-DA was performed to validate the significant differences between the group of isolated TPOAb-positive euthyroid pregnant women and the healthy control group (**Figure 1A**). During model validation, R2 was (0.0, 0.478), and Q2 was (0.0, 0.7318) (**Figure 1B**) and they suggested that this model was stable and reliable.

Differential lipid molecules were subjected to hierarchical clustering analyses (**Figure 1C**). The results showed clustering in some areas, with green representing the isolated TPOAb-positive group and red representing the control group. This indicated that the distribution of lipid metabolites differed between the two groups and provided a comprehensive overview of the lipid metabolite differences between the isolated TPOAb-positive pregnant women with normal thyroid function and the control group.

A total of 1238 lipid molecules were identified in our study. These molecules belong to 5 lipid categories (fatty acyl (FA), glycerophospholipid (GP), glycerolipid (GL), sphingolipid (SP), and sterol lipid (ST)) (shown in **Supplementary Figure S1A**). The number of lipids was greatest in the TG group. However, TG levels were not significantly different between the isolated TPOAb-positive pregnant group and the healthy control group (shown in **Supplementary Figure S1**). LPC levels were significantly increased in the isolated TPOAb-positive pregnant group (shown in **Supplementary Figure S1**).

The volcano plot used log2(FC) as the X-axis and -log10 (p-value) as the Y-axis. A total of 202 differentially abundant metabolites were screened in the TPOAb-positive euthyroid group, of which 135 were upregulated and 67 were downregulated (**Figure 1D**). The top 10 significant increases in lipid molecules were LPC (20:2e), AccA (20:4), LPC (22:2), LPC (16:0e), GM3 (m18:0/18:0), MLCL (10:2/18:0/22:0), LPC (18:0), LPC (20:1e), MLCL (14:2/18:0/18:0), and LPC (16:1e).

### 3.3 Pathway analysis of the differentially abundant metabolites

**Figure 2:**
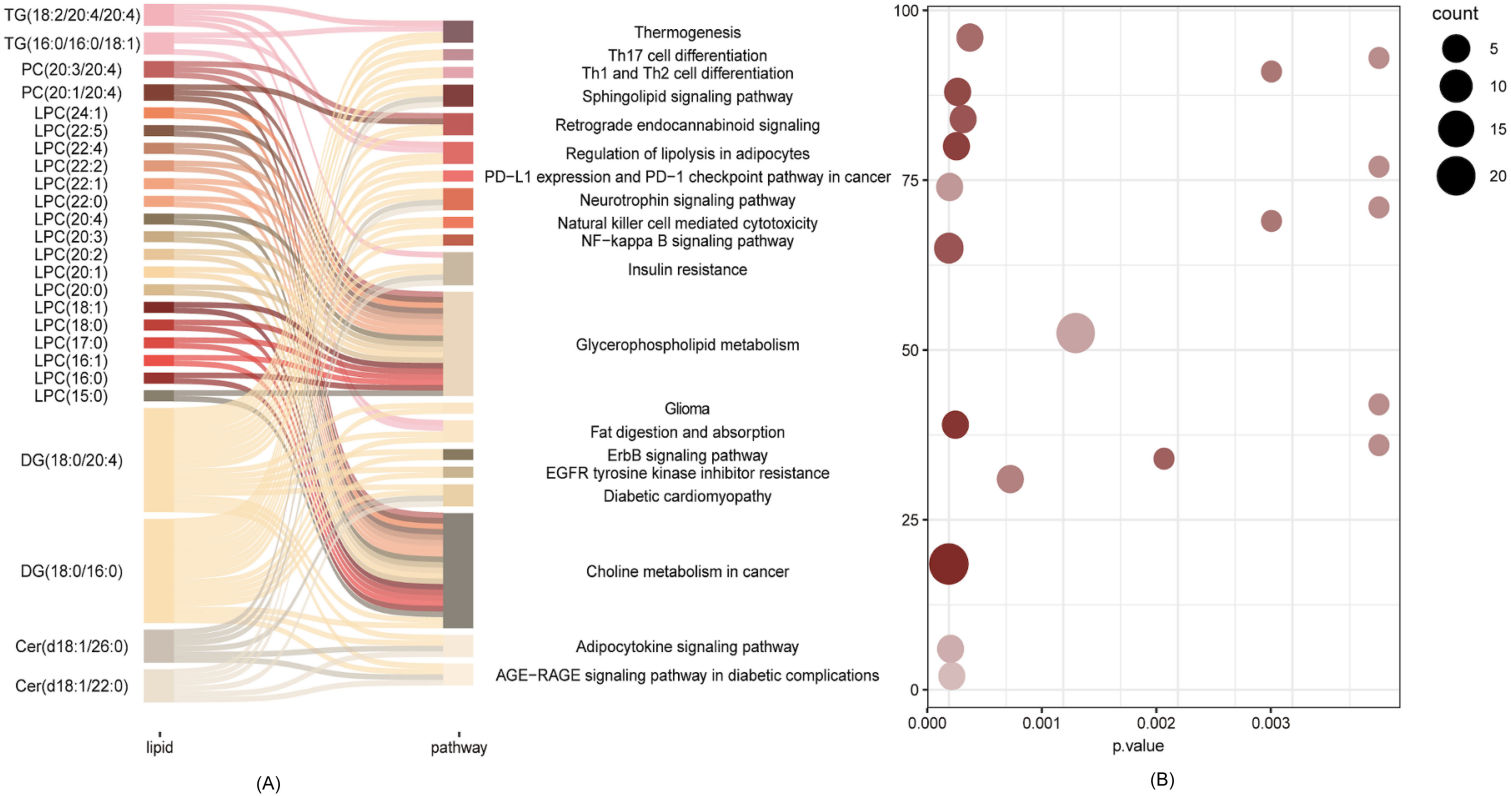
Pathways analysis of the differential lipid metabolite. **(A)** Network of differential lipid metabolites involved in the Choline metabolism in cancer, Glycerophospholipid metabolism, Insulin resistance, Adipocytokine pathway. **(B)** Scatter plot of KEGG enrichment3.

Enrichment analysis based on KEGG database was used to link the differential lipid metabolites to metabolic pathways. The results indicated that differential lipids in the isolated TPOAb-positive euthyroid pregnant women participated in many pathways, such as Thermogenesis, Sphingolipid signaling pathway, Regulation of lipolysis, PD-L1 expression and PD-1 checkpoint pathway in cancer, Neurotrophin signaling pathway, Natural killer cell mediated cytotoxicity, NF-kappa B signaling pathway, Insulin resistance, Glycerophospholipid metabolism, Choline metabolism in cancer, Adipocytokine signaling pathway, Fat digestion and absorption, AGE-RAGE signaling pathway in diabetic complications, Retrograde endocannabinoid signaling, Glioma, ErbB signaling pathway, EGFR tyrosine kinase inhibitor resistance, Diabetic cardiomyopathy, and the Th1/Th2/Th17 cell differential pathway (**Figure 2**).

Enrichment analysis showed that a total of 25 significantly different lipid molecules participated in the top enriched KEGG pathways. Except for Cer(d18:1/22:0); Cer(d18:1/26:0); DG(18:0/16:0); PC(20:1/20:4); PC(20:3/20:4); and TG(16:0/16:0/18:1), which were downregulated in the isolated TPOAb-positive euthyroid pregnant woman group, the other 19 differential metabolites were significantly upregulated (showed in **Figure 1D**). Many altered LPC molecules were closely involved in Glycerophospholipid metabolism and Choline metabolism in cancer pathways. Additionally, diacylglycerol (DG) (18:0/20:4) and DG (18:0/16:0) seem to act as bridges between pathways that require multiple metabolic pathways, connecting all the top enriched KEGG pathways except Glycerophospholipid metabolism (**Figure 2**).

### 3.4 Correlation analysis

**Figure 3:**
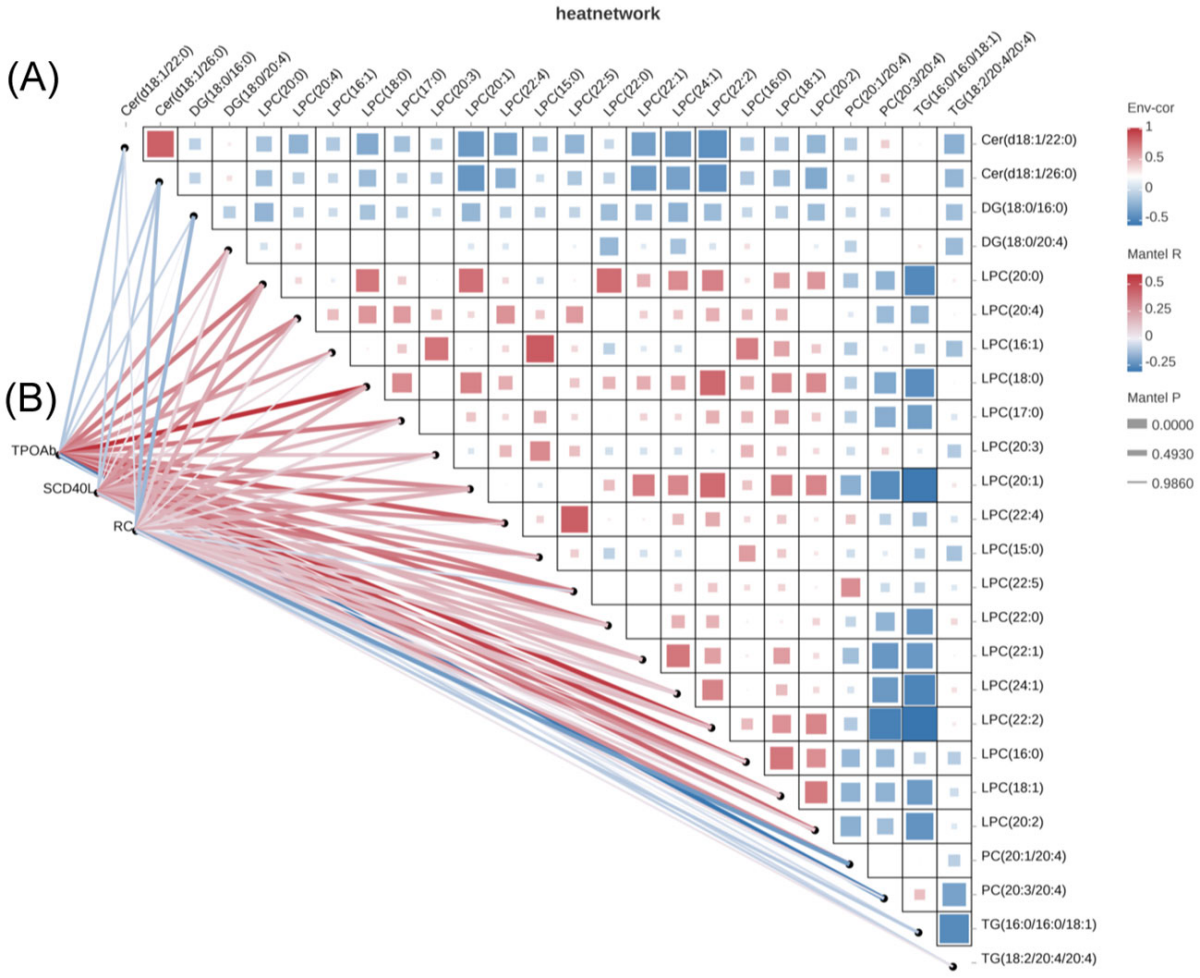
Correlation analysis of potential biomarkers and clinical parameters. **(A)** Potential biomarkers were selected based on the pathway enrichment analysis **(B)** Correlation analysis between potential biomarkers and clinical data.

Correlation analysis revealed that DG(18:0/20:4), LPC(20:0), LPC(20:4), LPC(16:1), LPC(18:0), LPC(17:0), LPC(20:1), LPC(22:4), LPC(22:5), LPC(22:0), LPC(22:2), LPC(16:0), LPC(18:1), LPC(20:2) were positively correlated with the TPOAb titer, while PC(20:3/20:4) was negatively correlated with the TPOAb titer. LPC (20:4), LPC(18:0), LPC(22:4), LPC(22:5), LPC(18:1), PC(20:1/20:4) were positively correlated with sCD40L levels (showed in **Figure 3**). LPC(20:0) was positively correlated with RC and PC(20:1/20:4) was negatively correlated with RC. No significant correlation was found between these different lipid molecules and FT4 levels.

## 4 Discussion

In the present study, we investigated differences in lipid profiles between isolated TPOAb-positive pregnant women and healthy controls in early pregnancy using LC-MS. We found significant changes in 202 lipid metabolites in isolated TPOAb-positive pregnant women compared with healthy controls. Pathway enrichment analysis showed that altered lipids might participated in multiple pathways, including Thermogenesis, Sphingolipid signaling pathway, Regulation of lipolysis, PD-L1 expression and PD-1 checkpoint pathway in cancer, Neurotrophin signaling pathway, Natural killer cell mediated cytotoxicity, NF-kappa B signaling pathway, Insulin resistance, Glycerophospholipid metabolism, Choline metabolism in cancer, Adipocytokine signaling pathway, Fat digestion and absorption, AGE-RAGE signaling pathway in diabetic complications, and the Th1/Th2/Th17 cell differential pathway. In particular, LPC(20:4), LPC(18:0), LPC(22:4), LPC(22:5), LPC(18:1) were positively correlated with both TPOAb titers and soluble costimulatory molecule sCD40L, indicating the intricate relationship between immune dysregulation and lipid metabolism.

Thyroid disease is a prevalent endocrine disorder encountered during pregnancy, and pregnant women who are positive for TPOAb may be at an elevated risk for thyroid dysfunction and adverse pregnancy outcomes (1–8). The fetal thyroid gland begins to function at approximately 20 weeks of gestation, but it is not fully functional until after birth. Therefore, there is an increased physiological demand for thyroid hormones during early pregnancy. Any disruption in maternal thyroid function can potentially affect the fetus, leading to developmental issues and an increased risk of adverse pregnancy outcomes. Due to the potential influence of TPOAb on thyroid function stability and subsequent adverse pregnancy outcomes, some prospective studies have explored the use of levothyroxine (LT4) intervention in isolated TPOAb-positive women during pregnancy to reduce adverse pregnancy outcomes. However, consistent results have not been obtained. Several randomized controlled clinical trials targeting TSH levels have shown that LT4 intervention during early pregnancy does not improve the incidence of isolated positive TPOAb in pregnant women with TSH levels <2.5 mIU/L (19, 20). In a prospective study reported by Negro et al., approximately 19% of women had TSH levels exceeding the upper limit of normal at delivery (21). Improved strategies or early biomarkers for detecting and managing thyroid dysfunction to reduce adverse outcomes during pregnancy are warranted.

Disruptions in lipid metabolism disorders are common features of thyroid dysfunction, and dysregulated lipidemia has been identified as a risk factor for adverse pregnancy outcomes (11–15). Consequently, lipid molecules in pregnant women with thyroid disease could serve as biomarkers for predicting SCH or CH or adverse pregnancy outcomes. Recently, lipidomics has emerged as a rapidly evolving field of science that concentrates on the investigation of the composition and function of lipids in the context of disease pathogenesis. According to the LIPIDMAPS classification, lipids are divided into fatty acids (FAs), glycerophospholipids (GPs), glycerolipids (GLs), sphingolipids (SPs), sterols (STs) and prenol lipids in humans (22). In our study, we identified 1238 lipid molecules and screened 202 differential lipids in TPOAb-positive pregnant women compared with healthy control. Notably, GPs, GLs and SPs were the most significantly altered lipid classes.

Glycerophospholipids (GPs) are pivotal constituents of cellular membranes that are integral to cellular signal transduction. Alterations in lipid metabolites in GPs have been linked to hypothyroidism, atherosclerosis, diabetes, insulin resistance and metabolic syndrome (23–25). It was suggested that TSH could result in the accumulation of GPs and affect thyroid metabolism in previous study (26). The GP family can be further classified into phosphatidylcholine (PC), phosphatidylethanolamine (PE), phosphatidylserine (PS), phosphatidylinositol (PI), phosphatidic acid (PA), and cardiolipin (CL). In hypothyroidism patients, Liu et al. reported an increase in the proportion of PCs, sphingomyelins (SMs), and PEs (24). An LC-MS study conducted by Li et al. on pregnant women with SCH revealed higher levels of PE, SM, and PC in the SCH group than in the healthy pregnant group. Notably, PC (40:7), PC (39:6), PC (38:4), PC (17:0/22:6), PC (17:0/20:4), and PC (20:1/22:6) were elevated in the SCH group (27). Cai et al. reported increased levels of SM (d36:1), PC (38:4), PE (36:4), PC (36:2), and PC (16:1/18:1) and PC (40:7) and decreased levels of SM (d42:6) and SM (d42:7), which might be potential biomarkers of hypothyroidism during pregnancy (28). In our study, we found a positive correlation between PE(8:0e/10:0) and TPOAb titers. PE is known to influence many cellular processes, including cellular stability and the functions of many membrane proteins, and is a crucial regulatory factor for cell membrane fluidity. Studies have indicated that PE was an essential stimulus of the Toll-like receptor (TLR) signaling pathway, which is associated with thyroid functions to further induce inflammation (23, 27, 29). Thus, PE might play an important role in the immunoinflammatory response associated with hypothyroidism during pregnancy. The significance of phospholipids such as PS and PE in the diagnosis of hypothyroidism should be further emphasized (30).

Lysophosphatidylcholine (LPC) seems to be a key lipid in our study. Many LPC molecules with different structures are significantly correlated with TPOAb titers. Additionally, these LPC molecules are involved in various pathways, such as glycerophospholipid metabolism, insulin resistance, and the adipocytokine pathway. Lysphospholipids, including LPC, lysophosphatidylinositol (LPI) and lysophosphatidyl ethanolamine (LPE), contain a FA moiety in their structures (22). LPCs are involved in various biological processes, including membrane signaling and cholesterol transport. Previous studies have demonstrated that LPC is a potent inflammatory mediator capable of activating various downstream signaling pathways, such as the MAPK and NF-κB pathways (31). LPCs act as endogenous ligands for G protein-coupled receptors (GPR132 and GPR4) and Toll-like receptors (TLR2 and TLR4), which are pivotal in initiating proinflammatory cascades. Activation of these receptors on immune cells, such as macrophages and T lymphocytes, triggers the release of cytokines and chemokines, fostering a microenvironment conducive to autoimmune activation (31, 32). In our study, we found that LPC(20:0), LPC(20:4), LPC(16:1), LPC(18:0), LPC(17:0), LPC(20:1), LPC(22:4), LPC(22:5), LPC(22:0), LPC(22:2), LPC(16:0), LPC(18:1), LPC(20:2) were significantly elevated in the isolated TPOAb-positive pregnant woman group, and these LPCs were positively correlated with TPOAb titers. These findings indicate that LPCs may be involved in the immune response that leads to the development of autoimmune thyroiditis. TPOAb are antibodies against thyroid peroxidase, an enzyme involved in the production of thyroid hormones, and their presence is indicative of an autoimmune response against the thyroid gland. It is well established that the activation of T cells and related cytokines contributes to the production of TPOAb (33, 34). Therefore, we speculate that the inflammatory environment brought about by the altered LPCs may play roles in the production of TPOAb.

CD40L is a membrane-bound protein expressed on T cells that interacts with CD40 on B cells, facilitating the differentiation, proliferation, and activation of B cells. Soluble CD40L (sCD40L), which is shed from the T-cell membrane, retains the ability to interact with CD40. The role of sCD40L is similar to that of membrane-bound CD40L (mCD40L) in that it activates CD40-expressing cells and facilitates the immune response (35). sCD40L has been found to be involved in various immune processes, including the regulation of antibody production, dendritic cell maturation, and the development of cellular immunity. It has also been implicated in the pathogenesis of certain diseases, such as systemic lupus erythematosus and rheumatoid arthritis, where increased levels of sCD40L have been observed (36). In our previous study, we demonstrated that sCD40L was positively correlated with TPOAb and associated with the risk of GDM and adverse neonatal outcomes in pregnant women with isolated positive TPOAb (7, 8). In addition, studies in the general population have found that the level of sCD40L levels are significantly increased in patients with hyperlipidemia, and sCD40L is not only involved in the inflammatory response but also in altering lipid levels by affecting the metabolism and transport of lipoproteins (37, 38). Notably, we found positive correlations between sCD40L and LPC(20:4), LPC(18:0), LPC(22:4), LPC(22:5), LPC(18:1), which were also positively correlated with the TPOAb titers in this study. These findings suggest that the altered lipid molecules observed in these isolated TPOAb-positive pregnant women might be a consequence of thyroid autoimmunity or that the altered lipid profile may contribute to the development of thyroid autoimmunity.

The emerging role of disturbed lipid metabolism in the development of hypothyroidism is an area of active research (13, 14). Based on an epidemiological study, Zhao et al. demonstrated that hypertriglyceridemia is associated with a greater risk of SCH and indicated that the thyroid might be an important target organ affected by lipo-toxicity (16). We explored the dysregulation profiles of lipid molecules in pregnant women with thyroid disease based on previous literature. Cai et al. reported that LPC (18:0) was significantly increased in pregnant women with hypothyroidism (28). Shao et al. also reported that LPC (18:0) and LPC (20:0) were elevated in pregnant women with hypothyroidism, but no significant difference in lipid metabolism was found between pregnant women with SCH and pregnant women with CH (13). The fact that LPC (18:0) is elevated in pregnant women with isolated TPOAb positivity, SCH, or CH suggests that this lipid may be an early predictive biomarker for the progression from autoimmunity to clinical or subclinical hypothyroidism (shown in **Supplementary Figure S2**). However, further studies are needed to confirm the relationship between altered lipid molecules and thyroid function in pregnant women.

Remnant cholesterol (RC) refers to the cholesterol content present within triglyceride-rich lipoproteins. Both epidemiological and genetic investigations have concurred that increased RC levels, irrespective of apolipoprotein B and LDL-c concentrations, are strongly associated with obesity, nonalcoholic fatty liver disease and metabolic syndrome (39–41). Furthermore, Sun et al. has demonstrated a close correlation between high fasting RC levels and the progression of thyroid dysfunction (42). We found that RC was significantly higher in isolated positive TPOAb pregnant women. Additionally, we found that LPC(20:0) was positively correlated with RC and PC(20:1/20:4) was negatively correlated with RC. This finding again links dyslipidemia to thyroid autoimmunity, in which lipid molecules may act as biomarkers or mediators of immune disorders, and the underlying mechanism still needs further investigation.

Our study has several strengths. First, we concentrated on pregnant women with a special thyroid disease phenotype, namely, TPOAb-positivity and euthyroid status, and preliminarily screened for lipid metabolism using LC-MS. Second, we proposed that 25 differential potential lipid molecules might participate in many pathways associated with adverse pregnancy outcome in euthyroid TPOAb-positive pregnant women. Finally, by comparing our data with those of previous studies, we found that some lipid molecules such as LPC (18:0) overlapped with that of pregnant women with SCH and CH, indicating the intricate relationship between lipid metabolism, thyroid autoimmunity, and thyroid function. This study has several limitations. Firstly, the sample in this study was relatively small due to the strict exclusion criteria, and our findings need to be further verified in larger studies. Secondly, this study employed a cross-sectional design, which limits ability to establish causality or observe longitudinal changes in lipid profiles during pregnancy. Lastly, this study focused on describing lipid metabolic changes but did not explore the underlying biological mechanisms linking lipids, immunity and thyroid function, and lipid metabolism, which need to be further verified.

## 5 Conclusion

In summary, we found the lipid profiles were significantly different in isolated TPOAb-positive pregnant women compared to healthy control. The altered lipid molecules participated in numerous pathways such as glycerophospholipid metabolism, insulin resistance, the adipocytokine pathway, and Th1/Th2/Th17 cell differentiation which might also be associated with the related adverse outcome in pregnant women with thyroiditis. Additionally, positive correlation was observed between these differential lipid molecules and both TPOAb antibodies and soluble costimulatory molecules. The pathogenetic role of differential lipid metabolites during pregnancy should be further investigated.

## Data Availability

The raw data supporting the conclusions of this article will be made available by the authors, without undue reservation.

## Supplementary material

Supplementary Figure 1 Cluster analysis of differential lipids **(A)** 1238 lipid molecules belonging to 5 lipid categories (fatty acyl (FA), glycerophospholipid (GP), glycerolipids (GL), sphingolipid (SP), sterol lipid (ST)) were identified. The comparison of lipid molecular species for the isolated TPOAb positivity euthyroid pregnant women and control group (represented in the green and red color respectively) was showed in FA **(B)**, SP **(C)**, GP **(D)**, GL **(E)**, respectively.

Supplementary Figure 2 Overlapping between dysregulated lipids profile in pregnant women with thyroid disease. The differential lipids were compared with previous literatures and found LPC (18:0) were both upregulated in euthyroid with isolated positive TPOAb, subclinical hypothyroidism and clinical hypothyroidism during pregnancy.

## Glossary

TPOAb: thyroid peroxidase antibody
LC-MS: liquid chromatography-mass spectrometry
FA: fatty acyl
GP: glycerol phospholipid
GL: glycerol ester
SP: sphingolipid
ST: sterol lipid
KEGG: Kyoto Encyclopedia of Genes and Genomes
sCD40L: soluble CD40 ligand
LPC: lysophosphatidylcholine
TgAb: thyroglobulin antibody
GDM: gestational diabetes mellitus
TG: triglyceride
TC: total cholesterol
LDL: low density lipoprotein
HDL: high density lipoprotein
RC: remnant cholesterol
HL: hepatic lipase
CEPT: cholesterol transfer protein
LDL-c: low density lipoprotein cholesterol
SCH: subclinical hypothyroidism
CH: clinical hypothyroidism
HT: Hashimoto thyroiditis
FT4: free thyroxine
TSH: thyrotropin
BMI: body mass index
HbA1c: glycosylated hemoglobin A1c
UA: uric acid
CRP: C-reactive protein
WBC: white blood cell
RBC: red blood cell
Hb: hemoglobin
PLT: platelet
UPLC: ultrahigh-performance liquid chromatograph
MTBE: methyl tertiary butyl ether
QC: quality control
ISVE: ion-spray voltage floating
DDA: Data Dependent Acquisition
RT: retention time
m/z: mass-to-charge ratio
RSD: relative standard deviation
PCA: principal component analysis
PLS-DA: supervised partial least squares-discriminant analysis
VIP: Variable importance in the projection
FC: fold change
GM3: monosialodihexosylganglioside
MLCL: monolysocardiolipin
DG: diacylglycerol
LdMePE: lysodimethylphosphatidylethanolamine
MGDG: monogalactosyl diacylglycerols
ChE: cholesterol ester
PC: phosphatidylcholine
PE: phosphatidyl ethanolamine
PS: phosphatidylserine
PI: phosphatidylinositol
PA: phosphatidic acid
CL: cardiolipin
SM: sphingomyelin
LPI: lysophosphatidylinositol
LPE: lysophosphatidyl ethanolamine
