# Supplementary figures and images for: Differential lipids in euthyroid pregnant women with positive TPOAb and its correlation with clinical parameters
Source: Front Endocrinol (Lausanne). 2025 Mar 27;16:1433534. doi: 10.3389/fendo.2025.1433534 (PMC11982940; doi:10.3389/fendo.2025.1433534)

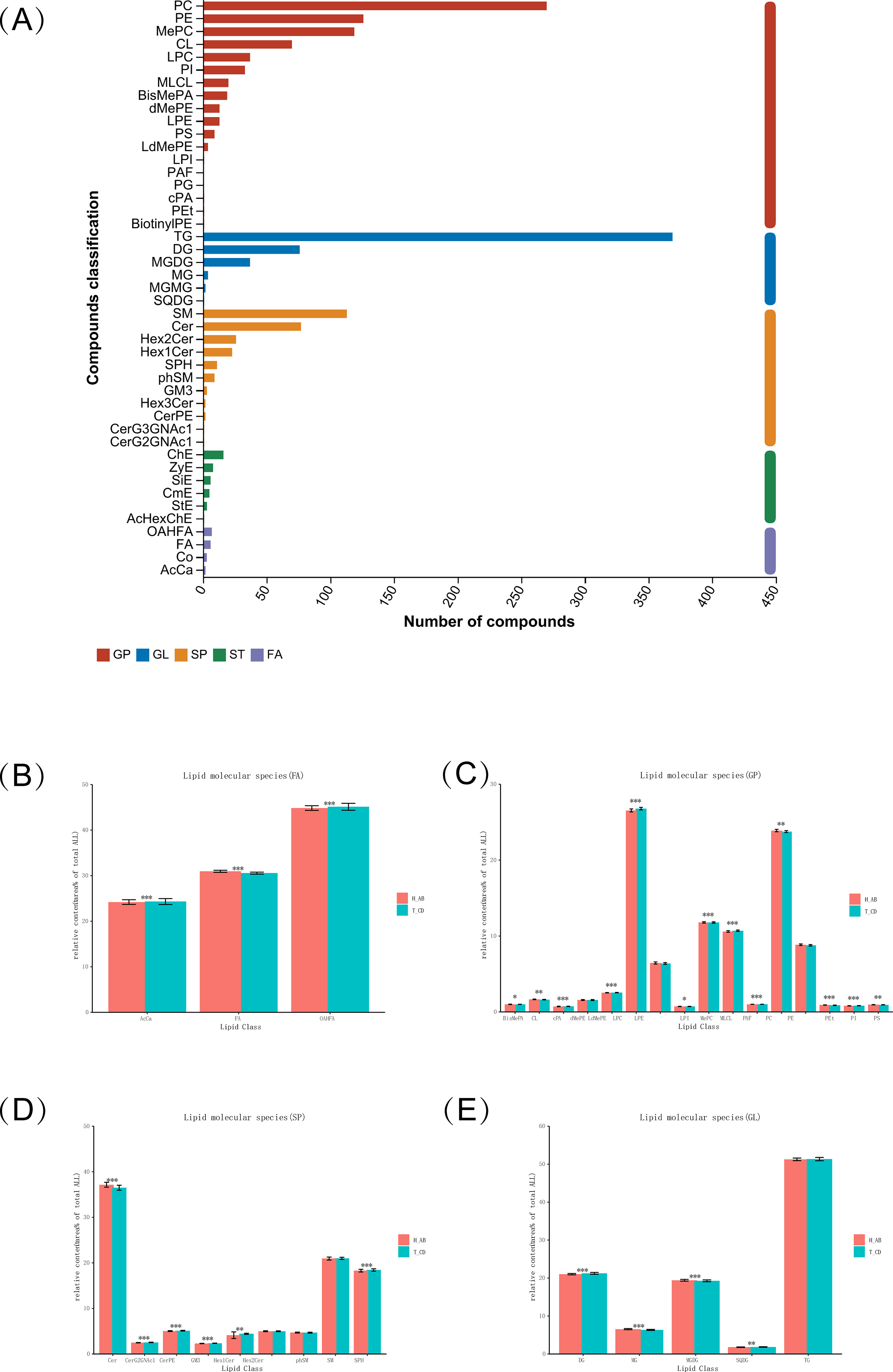

Supplement: Supplementary Figure 1 — Cluster analysis of differential lipids (A) 1238 lipid molecules belonging to 5 lipid categories (fatty acyl (FA), glycerophospholipid (GP), glycerolipids (GL), sphingolipid (SP), sterol lipid (ST)) were identified. The comparison of lipid molecular species for the isolated TPOAb positivity euthyroid pregnant women and control group (represented in the green and red color respectively) was showed in FA (B), SP (C), GP (D), GL (E), respectively. [file Image1.tif]
